# The German interprofessional attitudes scale: translation, cultural adaptation, and validation

**DOI:** 10.3205/zma001325

**Published:** 2020-04-15

**Authors:** Tina H. Pedersen, Eva Cignacco, Jonas Meuli, Ferdinand Habermann, Joana Berger-Estilita, Robert Greif

**Affiliations:** 1 University of Bern, Bern University Hospital, Inselspital, Department of Anesthesiology and Pain Therapy, Bern, Switzerland; 2 Bern University of Applied Sciences, Health Professions, Midwifery Division, Bern, Switzerland

**Keywords:** interprofessional attitudes, assessment, psychometric testing, transcultural translation

## Abstract

**Objectives: **The implementation of obstetric hybrid simulation and interprofessional collaboration between midwives and anesthetists in labor emergencies fostered the need to evaluate the impact of such a program. The original Interprofessional Attitude Scale (IPAS) assesses interprofessional attitudes among health professional students and includes the 2011 and 2016 Interprofessional Collaborative Practice report competency domains. The purpose of this study was to create a German version of the IPAS (G-IPAS) to use for the education of healthcare students.

**Methods: **We performed the translation and validation of the IPAS in five steps:

1. translation to German according to the International Society of Pharmaeconomics and Outcome Research guidelines;
2. nine cognitive interviews with healthcare professionals and students;
3. calculation of the Content Validity Index (CVI) by expert opinion;
4. exploratory factor analysis (EFA); and
5. internal consistency by Cronbach’s alpha.

All study participants gave written informed consent and the cantonal ethics committee waived further ethical approval.

**Results: **The cognitive interviews led to replacement of single-item wording. We retained 27 items for CVI analysis. The averaged overall CVI was 0.79, with 15 items ≥0.89. 185 students (70 medicine, 51 nursing, 48 physiotherapy, and 16 midwifery) contributed with data for the EFA and it produced three subscales. *“Teamwork, roles, and responsibilities”* with factor loadings ≥0.49, *“Patient-centeredness”* with factor loadings ≥0.31, and *“Community-centeredness”* with factor loadings ≥0.57. Two items of the total scale were deleted, and four items were redistributed to another subscale. Cronbach’s alpha for the overall G-IPAS scale was 0.87. After deleting and redistributing items in subscales, a new Scale-CVI/Average was calculated and was 0.82.

**Conclusions: **Based on a rigorous validation process, the G-IPAS provides a reliable tool to assess attitudes towards interprofessional education among different healthcare professions in German-speaking countries.

## Zusammenfassung

**Zielsetzung: **Die Implementierung von geburtshilflichen Hybrid-Simulationen und interprofessioneller Zusammenarbeit zwischen Hebammen und Anästhesisten bei Schwangerschaftsnotfällen macht es notwendig, den Einfluss eben solcher Programme zu untersuchen. Die ursprüngliche Interprofessional Attitude Scale (IPAS) bewertet die interprofessionelle Einstellung unter Studierenden der Gesundheitsberufe und umfasst die Kompetenzbereiche der Interprofessional Collaborative Practice Berichte von 2011 und 2016. Der Zweck dieser Studie war es, eine deutsche Version der IPAS (G-IPAS) zu erstellen, um sie für die Ausbildung von Studierenden in den Gesundheitsberufen zu nutzen.

**Methoden: **Wir haben die Übersetzung und Validierung des IPAS in 5 Schritten durchgeführt:

1. Übersetzung in das Deutsche anhand der Richtlinien der International Society of Pharmaeconomics and Outcome Research;
2. neun kognitive Interviews mit Experten und Studierenden aus den Gesundheitsberufen;
3. Berechnung des Content Validity Index (CVI) nach Experten Wertung;
4. explorative Faktorenanalyse (EFA); und
5. interne Konsistenz mittels Cronbach’s Alpha.

Alle Studienteilnehmer haben eine schriftliche Einverständniserklärung abgegeben und das Kantonale Ethikkomitee erteilte eine Ausnahmegenehmigung.

**Resultate: **Die kognitiven Interviews führten dazu, dass einzelne Items ersetzt wurden. Wir behielten 27 Begriffe für die CVI Analyse. Der durchschnittliche Gesamt-CVI betrug 0.79, mit 15 Items ≥0.89. 185 Studierende (70 Medizin, 51 Pflege, 48 Physiotherapie, und 16 Hebamme) haben die Daten für die EFA beigetragen und diese erzeugten drei Unterskalen. *„Teamarbeit, Funktionen und Verantwortlichkeiten“* mit einer Faktorauslastung von ≥0.49, *„Patientenzentriertheit“* mit einer Faktorauslastung von ≥0.31, und *„Gesundheitsversorgung“* mit einer Faktorauslastung von ≥0.57. Zwei Items der gesamten Skala wurden entfernt und vier Items wurden einer anderen Unterskala zugeordnet. Die Cronbach’s Alpha für die gesamte G-IPAS Skala beträgt 0.87. Nach dem Löschen und Umverteilen der Items in Subskalen wurde ein neuer Skala-CVI/Durchschnitt berechnet, welcher nun 0.82 ist.

**Schlussfolgerungen: **Basierend auf dem strengen Validierungsprozess bietet der G-IPAS ein zuverlässiges Instrument zur Bewertung der Einstellung zur interprofessionellen Ausbildung bei verschiedenen Gesundheitsberufen im deutschsprachigen Raum.

## Introduction

Interprofessional collaborative practice has become a landmark to address complex healthcare issues. Evidence indicates that skillful interprofessional education (IPE) fosters effective collaborative practice [1]. According to World Health Organization, IPE occurs when “students from two or more professions learn about, from, and with each other to enable effective collaboration and improve health outcomes” [2]. The Interprofessional Education Collaborative Expert Panel (IPEC) reported that safe, high-quality, accessible, patient-centered care requires continuous development of interprofessional competencies by students of different health professions as part of their learning process to enter workforce with skills for effective teamwork and team-based care [3]. 

The implementation of obstetric hybrid simulation and interprofessional collaboration between midwives and anesthetists in labor emergencies at Bern University Hospital, Switzerland fostered the need to evaluate the impact of such a program. Obstetric hybrid-simulation embraces actresses playing pregnant women giving birth, to provide a “close-to-real-life” learning situation. Participants practice interprofessional competence, partly under stress, without risk for the laboring woman and newborn. During video-assisted debriefing, participants share their experiences and hereby learn about each other’s professions, responsibilities, perspectives, and attitudes.

Until recently, a paucity of conceptual frameworks and tools existed for assessing IPE outcomes [4]. The Readiness for Interprofessional Learning Scale (RIPLS) [5] and the extended RIPLS [6] are established tools assessing interprofessional (IP) attitudes with translations in several languages [7], [8], [9], [10] applied in different cultural contexts. For the German speaking countries exists a German version of the University of the West of England Interprofessional Questionnaire (UWE-IP) [11]. These scales were developed before the 2011 *Core Competencies for Interprofessional Collaborative Practice (IPEC)* report and fail to embody all four recommended IP core-competency domains: values/ethics for IP-practice; roles/responsibilities; IP-communication; and teams/teamwork [3]. A new scale was developed and validated in 2015, the Interprofessional Attitudes Scale (IPAS) [12], using items from the extended RIPLS and new items to cover all four IPEC-report competency domains. The updated IPEC report on *Core Competencies for Interprofessional Collaborative Practice* from 2016 does still have the same four core competencies [13]. 

The IPAS has 27 survey-questions that load into 5 subscales:

1. teamwork, roles, and responsibilities (TRR);
2. patient-centeredness (PC);
3. interprofessional bias (IB);
4. diversity and ethics (DE) and
5. community-centeredness (CC) [12].

Currently, no German version of the IPAS exists. Using the same tool in different countries may provide opportunities for international research in order to corroborate further knowledge acquired in IPE [14]. The purpose of this study was to translate the English IPAS into German and perform psychometrical analysis to have a validated tool for the assessment of interprofessional attitudes.

## Methods

**Figure 1:**
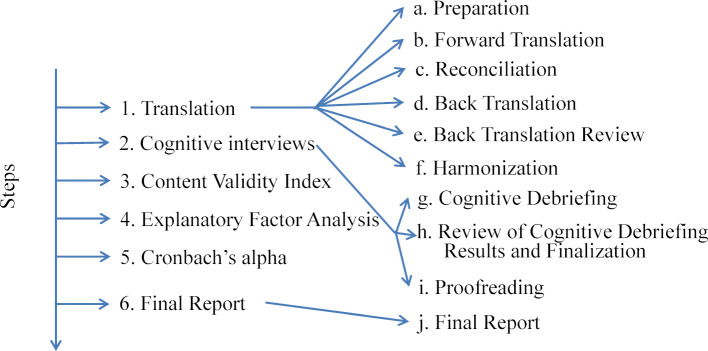
Steps of the study, Bern, 2016

To establish a German IPAS (G-IPAS) we looked to the principles recommended by the International Society of Pharmaeconomics and Outcome Research (ISPOR) [15] for the translation and the cultural adaptation (see figure 1 (Fig. 1)):

1. Translation of the English IPAS into German,
2. Cognitive interviews to rephrase or delete items in the German version, if they were not comprehensible or relevant to potential users,
3. Validity established by the Content Validity Index (CVI),
4. Exploratory factor analysis (EFA) to uncover the underlying structure of items and create meaningful subscales, and
5. Cronbach’s alpha calculation for single items, subscales, and the whole scale to assess internal consistency

Because validation is not part of the ISPOR guidelines, we added a validity analysis between stage I and J: this included a content validity analysis using the Content Validity Index (CVI); an exploratory factor analysis to uncover the underlying structure of the items and create meaningful subscales, and we calculated Cronbach’s alpha for assessment of internal consistency. 

### Step 1: Translation of the original IPAS 

After obtaining permission from the authors, the English IPAS was translated and harmonized by five native German speakers from Germany, Switzerland, and Austria with health care background [12]. The five translations were merged into a single version in a nominal group discussion. The nominal group technique takes advantage of pooled judgments. That means that the judgments of a variety of people with varied talents, knowledge, and skills will be used together. By doing this, the resulting ideas are likely to be better than those that might be obtained by other methods [16], [17]. This merged G-IPAS was then translated back into English by a native English speaker. The original American version, the version translated back into English, and the German version were then compared and harmonized to ensure the conceptual equivalence between the different IPAS versions in another nominal group discussion. The final harmonized G-IPAS version was then proofread before it was used in the cognitive interviews.

#### Step 2: Cognitive interviews

Covering step G to I of the ISPOR guidelines (see figure 1 (Fig. 1)) [15], the G-IPAS was pre-tested among nine healthcare professionals and students. We conducted cognitive interviews with three bachelor’s degree students from the midwifery program of the University of Applied Sciences Bern, three certified registered anesthesia nurses, and three anesthesia residents of the Department of Anesthesiology and Pain Therapy, Bern University Hospital in Switzerland. All participants had experience in interprofessional teaching and simulation. Cognitive interviews intensively probe the thought processes of individuals who are presented with those inquiries and help researchers discover how well their questions are working, where they are failing, and determine how they can improve [18]. The interview goal was to rephrase or delete items from the G-IPAS, if items were not comprehensible or relevant to potential users. All participants were asked two questions about every item in the G-IPAS:

1. *“Can you repeat the item in your own words?”*, and
2. *“What is your understanding of this item?”*.

Two members of the study group (TP medical doctor, JM research associate) recorded the interviews, debriefed the results orally, and adjusted the items according to the results. After proofreading, the German IPAS was ready for validation.

#### Step 3: Content validity of the translated G-IPAS

After the cognitive interviews, we calculated a Content Validity Index (CVI) for each item and for the whole scale using expert opinion [19]. We asked nine health care providers with experience in interprofessional teaching and simulation (three midwives, three anesthesia nurses,and three consultants in anesthesia, all nine with >10 years of experience) to rate the relevance of each item on a scale from 1-4, with 1=not, 2=somewhat, 3=quite, and 4=highly relevant. The agreement among experts was assessed by calculating the Item Content-Validity Indexes (I-CVI). The I-CVI computes by the number of experts giving a rating of 3 or 4, divided by the total number of experts. Items with an I-CVI >0.78 are considered having excellent content validity, whereas items ≤0.78 needs to be revised [20]. We assessed the validity of the entire questionnaire with the averaged I-CVI across all items, called Scale-CVI/Average (S-CVI/Ave). An S-CVI ≥0.8 is acceptable [21], [22] and ≥0.90 means excellent content validity [23]. 

#### Step 4: Exploratory factor analysis

**Figure 2:**
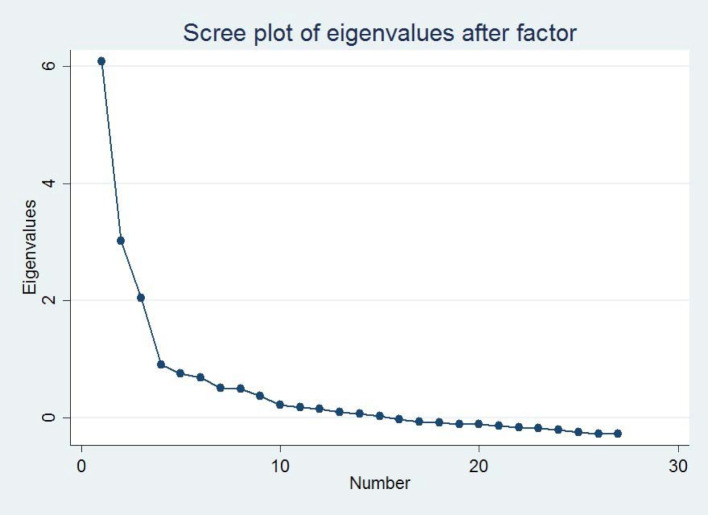
Scree plot of Eigenvalues, Bern, 2016

We asked medical, nursing, physiotherapy, and midwife students from the University of Bern and the University of Applied Sciences of Bern to fill out the G-IPAS after class. The EFA intends to uncover the underlying structure of the items. We followed Osborne/Costello’s recommendations [24] using principal axis factoring (PAF) for non-parametric data. The correlation matrix was inspected for evidence of coefficients greater than 0.3, indicating strength of the intercorrelation among items. We tested the sampling adequacy for factor analysis using the Kaiser-Meyer-Olkin (KMO) measure of sampling adequacy [25]. A KMO index of 0.6 was the accepted minimum value for a good factor analysis [26]. We performed a scree test [27] to decide the number of factors to retain (see figure 2 (Fig. 2)). Factors were extracted based on eigenvalues >1 [25]. Finally, we conducted a Direct Oblimin rotation to assure a more accurate and reproducible factor solution [24]. 

#### Step 5: Assessment of internal consistency (Cronbach’s alpha and item total correlation)

After performing the EFA, we tested the internal consistency of the instrument by calculating Cronbach’s alpha for single items, for subgroups, and for the final scale as a whole. We reversed negatively formulated items before checking internal consistency. An alpha value of >0.70 was regarded as satisfactory [28], [29]. We also calculated the item total correlation to show how highly correlated each item is with the overall scale. An item should correlate with the total score above 0.3, but not above 0.7 [30]. 

Stata/SE 14.1 (Stata Corp. LP, College Station, TX, USA) analyzed all data. 

## Results

### Step 1: Translation of the original IPAS 

The original IPAS word count is about 2,500 characters including spaces, while the G-IPAS ended up with about 3,500 characters. In correspond to the English version, G-IPAS entailed five dimensions and 27 items in total after translation. 

#### Step 2: Cognitive interviews

After the first six interviews, items were adjusted according to comments from the interviewees. The remaining three interviews led again to re-adjustment of items. The input from the cognitive interviews led to replacement of single-item wording (e.g. *“Empathie (empathy)” instead of “Mitgefühl (sympathy)”* in patient-centeredness (PC2) (see attachment 1 for the English and German items). Item wording was shortened: e.g. *“Vertrauen (trust)”* instead of *“Vertrauensverhältnisses (relation of trust)”* in (PC1), and *“Rollen (roles)”* instead of *“Rollenverständnis (role understanding)”*. In total, 16 out of 27 items underwent a word change based on the cognitive interviews. Interviewees questioned the relevance of some items in European healthcare context, especially for the dimension* “Community-centeredness”* (e.g. item CC3 *“It is important for health professionals to work with legislators to develop laws, regulations, and policies that improve health care”*). All 27 items were retained for further CVI. 

#### Step 3: Content validity of the translated G-IPAS

The G-IPAS average content validity index with all 27 items is 0.79. 15 items (56%) had an I-CVI ≥0.89 (see table 1 (Tab. 1)). Eight items had an I-CVI between 0.56 and 0.78, and four items had an I-CVI ≤0.44. Before we deleted items with low CVI, we performed EFA to test the underlying structure of G-IPAS to have a sound basis to delete or retain items.

#### Step 4: Exploratory factor analysis

For EFA and internal consistency testing, 185 students (70 medicine, 51 nursing, 48 physiotherapy, and 16 midwifery) filled in the questionnaire with a 100% response rate (see table 2 (Tab. 2), Demographic data). 

The 27 items of the G-IPAS were subject to principal component analysis (PCA) [21]. Prior to PCA, we assessed the suitability of data for factor analysis. Inspection of the correlation matrix revealed many coefficients of 0.3 and above. The KMO value was 0.82, exceeding the recommended value of 0.6 and supporting the factorability of the correlation matrix.

We used scree plot for factor extraction, which showed three data points above the break, and we retained three factors (see figure 2 (Fig. 2)). These three factors were the only factors with an eigenvalue >1 (see attachment 1 , displaying Eigenvalues and variances). 

All nine items in dimension *“Teamwork, roles, and responsibilities”* had factor loadings ≥0.49 on factor 1. Further analysis of the rotated solution in the pattern matrix is presented in attachment 1 . Items in the dimension *“Patient-centeredness”* had factor loadings ≥0.31 on factor 3 (five items). All six items in the dimension* “Community-centeredness”* had factor loadings ≥0.57 on factor 2. For the dimension *“Interprofessional bias”*, the item IB1 did not have loadings above 0.30 on any factors. Item IB2 loaded negatively on factor 3, but was not negatively formulated. Item DE1 in the dimension *“Diversity and Ethics”* loaded on factor 2 with 0.39. The three other items in *“Diversity and Ethics”* loaded on factor 3. 

#### Decision to keep or delete items 

IB1, IB2, and IB3 were deleted based on low CVI and EFA results. IB1 and IB2 had low I-CVI’s of 0.44 and 0.56 and neither had loadings >0.30 on any of the three factors in the EFA. We deleted IB3 because of low I-CVI (0.67). We integrated the rest of the items into three groups based on which factor they loaded on. 

TRR1-TRR9 stayed together in the dimension *“Teamwork, roles and responsibilities”*. 

DE2-4 were assembled with PC1-5 in the subgroup *“Patient-centeredness”*. 

DE1 was assembled with CC1-6 in a new dimension called *“Healthcare Provision”* (see attachment 2 : Final German-IPAS). 

After deleting and redistributing items in subgroups, a new S-CVI/Ave was calculated. The new value was 0.82 (see table 1 (Tab. 1)).

#### Step 5: Cronbach’s alpha and item total correlation

The IPAS scale had moderate to good internal consistency (Cronbach’s alpha coefficient between 0.62 and 0.92) [12]. 18 items had a value between 0.30 and 0.70, five above 0.70 and only one item had a value of 0.26 (see table 3 (Tab. 3)). The overall G-IPAS Cronbach’s alpha after deleting and redistributing of items was 0.87, showing very good internal consistency

## Discussion

We have described the translation of the original American Interprofessional Attitudes Scale (IPAS) into German. The translated G-IPAS shows good reliability and replicated the factor structure of the original IPAS version. Therefore, it can be recommended for the use in German-speaking countries. Furthermore, G-IPAS shows similar internal consistency when compared to the original version [12]. Although the factor structure was replicated, high correlation between individual items was found, indicating that these items may not represent different dimensions.

The original IPAS was based on RIPLS and extended RIPLS [6], whose psychometric integrity for measuring interprofessional education has been criticized [12], [31], [32], [33]. However, IPAS shows consistent improvements over RIPLS regarding psychometric characteristics. RIPLS was criticized for its *evidence for validity* because students did not have any direct input to the development of the instrument; in contrast students and faculty developed IPAS. For the cultural adaptation process of the G-IPAS, we invited health care providers and students from several German-speaking countries, thereby enhancing cultural adaptation and ensuring that the perspectives of users and issues relevant to an interprofessional training were captured by G-IPAS. Additionally, RIPLS did not report the relationship between the construct and outcome being measured. In our German translation and cultural adaptation, we have performed such “think aloud” interviews with the cognitive interviews to overcome that limitation. Additionally, both RIPLS and the original IPAS have subscales with Cronbach’s alpha below 0.70, while the G-IPAS does not, which is another hint that the cultural adaptation worked properly for G-IPAS. 

RIPLS did not report reliability information [31]. External *evidence for reliability* is not applicable to the IPAS, since it is not measuring a “stable” phenomenon. We assessed internal reliability for G-IPAS with backward and forward translation, cognitive interviewing, CVI, EFA, and Cronbach’s alpha. The described internal reliability provides sufficient homogeneity of the G-IPAS and its items to make sure that the measurement of interprofessional attitudes in the German-speaking countries is understandable and makes sense to its user. We ensured that the adopted items of the G-IPAS really do measure what is intended, and that the single items of G-IPAS are built up in a coherent way to measure interprofessional attitudes.

The cultural adaptation was important to sharpen the wording for a more precise German understanding. To validate our cultural adaptations in this translation, we calculated I-CVI and S-CVI [19]. Four original IPAS items scored low as these items were not well adapted to the German-speaking healthcare environment. That might explain why the average content validity index was 0.82, slightly below the recommended average of 0.90.

CVI together with EFA sharpened the cultural adaptation, by deleting items that made no sense in the central European health care environment. Interestingly, in this EFA all nine items in the subscale *“Teamwork, roles and responsibilities”* loaded on factor 1, all five items in the subscale *“Patient-centeredness”* loaded on factor 2, and all six items in the subscale *“Community-centeredness”* loaded on factor 3 (see attachment 1 ). This reinforced us to keep these subscales in the G-IPAS. In contrast, we found very low loading and double loading on factors in *“Interprofessional Bias”* and *“Diversity and Ethics”*. That called for better cultural adaptation for the German-speaking area of healthcare.

All items in the subscale* “Interprofessional Bias” *scored low in I-CVI (see table 1 (Tab. 1)), and loaded on the same factor in the EFA. As this subscale only has three items but should have at least 5 factors [24] and reached a lower Cronbach’s alpha in the original IPAS compared to other subscales, the question arose if this subscale should stay in the G-IPAS. The authors of the original IPAS kept it *“because the attitudes it assesses impact several IPEC Report core competencies”* [12]. Going through the IPEC Report and its core competencies, the words *“prejudice”, “assumptions”, “judgement”, “bias”*, or *“tendentious”* do not appear [3]. As these core words to assess interprofessional attitudes were not directly mentioned in the report, we found it reasonable to delete the entire subscale from G-IPAS.

The items from *“Diversity and Ethics”* did not consistently load on only one factor. Based on factor loading we distributed these items to the three remaining subscales. Items DE2, DE3, and DE4 are patient related (communication across cultures, respecting the privacy of patients, providing equal treatment despite background). As they loaded on the same factor as items in *“Patient-centeredness”*, we allocated them to *“Patient-centeredness”*. Item DE1 (respecting other health professions) loaded on the same factor as items in the subgroup *“Community-centeredness”*, and we placed DE1 in that subgroup, as the addition of that extra item made the subgroup more solid [24]. Because of all these results from the cultural adaptation, we renamed the subscale *“Community-centeredness”* to *“Health Care Provision”*. 

Finally, a Cronbach’s alpha of 0.87 for the whole scale provided satisfactory internal consistency of the new G-IPAS (*“Teamwork, roles and responsibilities”* scored 0.88, *“Patient-centeredness”* 0.78, *“Health care provision”* 0.85). The item total correlation reconfirmed that G-IPAS is a valid instrument, as 18 items had a score of 0.30 to 0.70. Only one item correlated below 0.30 (PC1 “*Establishing trust with my patients is important to me”*). We did not delete it, because establishing trust with patients seems to be an important competence in IPE. The five items with an item total correlation above 0.70 (TRR2, TRR3, TRR7, HCP2, HCP3) were kept too, as these questions are essential to assess interprofessional attitudes.

G-IPAS has some limitations. RIPLS was criticized for not having validity evidence based on relationship to other variables, meaning the degree to which the score of an instrument correlates to scores obtained by others, for example, if the instrument measures outcomes of IPE as directly observed by an assessor. The original IPAS and the G-IPAS also have this limitation. As intended and unintended consequences of an instrument’s use are relevant to its applicability, G-IPAS ratings from students of IPE events must be analyzed to extract such evidence. 

Our EFA sample size was not identical to the original IPAS and this might have influenced our results. A larger sample size definitively increases the confidence in the factor analysis results and the power to detect significant changes among the constructs that were measured. However, the subject to item ratio was 6:1, which is considered adequate for factor analysis [26] and the use of self-report instruments is a challenge when measuring interprofessional outcomes [11]. 

We did not perform a confirmatory factor analysis (CFA). In our opinion the CFA comes as a later step that we can perform when we have used the G-IPAS and collected further data. 

## Conclusion

The original American IPAS with five subscales was translated, culturally adapted, and validated, hereby creating the German IPAS (G-IPAS). This validation process led to the deletion of the subscale *“Interprofessional Bias”* and re-distribution of items from the subscale* “Diversity and Ethics”* to the remaining three subscales: *“Teamwork, Roles and Responsibilities”*, *“Patient-centeredness”* and the renamed subscale *“Health Care Provision”*. 

The G-IPAS is a reliable instrument, which appropriately represents the items of the original IPAS, and is a validated tool for the assessment of interprofessional attitudes in interprofessional education and interprofessional training to be used in German-speaking countries. 

## Acknowledgements

The authors acknowledge the help of Lorenz Theiler, Maren Kleine-Brueggeney, Maximillian Buttenberg, Tobias Hornshaw, and Simon Fisher in the translation of the IPAS to German. The authors would also like to thank Isabelle Romano, Ines Uhr, Dorothée Eichenberger zur Bonsen, Christine Riggenbach, Mathias Scherz, Yves Balmer, Thomas Arnold, and Stefan Lötscher for their input for the CVI. Finally, the authors would like to thank all the students from Bern University for Applied Sciences and University of Bern for participating in the study. 

## Ethical approval

The Cantonal Ethics Committee of Bern reviewed the study protocol (“Req-2016-00176/ 12.04.2016”). The Ethics Committee granted a waiver for the study as that research project does not fall under the Swiss Human Research Act (Art. 2, Abs.1). All study participants gave written informed consent before participating in this study.

## Funding

A departmental research grant dedicated to Prof. Robert Greif sponsored this study.

## Previous presentations

A preliminary report was presented in poster form at the Congress for Health Care Professionals “Interprofessionalität – Realität oder Mythos?” 4 March 2016. Preliminary results were accepted for a poster presentation at the 31st ICM Triennial Congress June 2017, Toronto, Canada. Preliminary results were presented in an oral presentation at SPSIM Congress, March 2017 Bern. 

## Competing interests

The authors declare that they have no competing interests. 

## Erratum

An affiliation was corrected. 

## Supplementary Material

Factor loading results of an exploratory factor analysis of the German Interprofessional Attitudes Scale, Bern, 2016

Final version German IPAS

## Figures and Tables

**Table 1:**
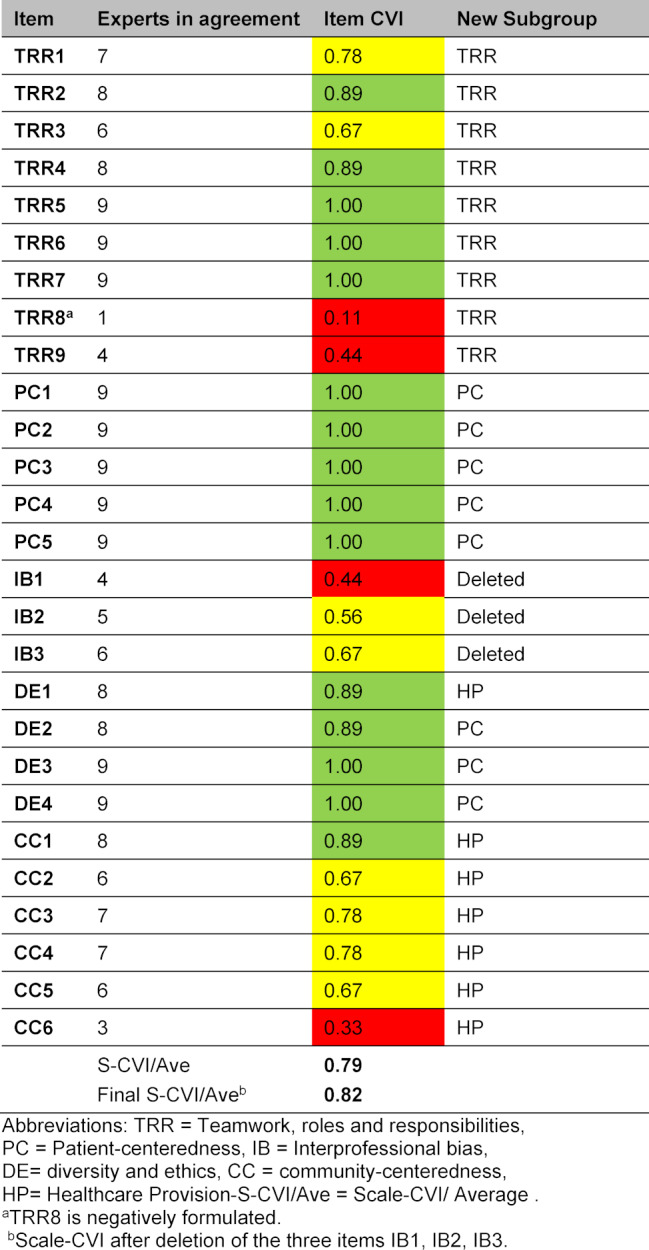
Content validity index (CVI), Bern, 2016

**Table 2:**
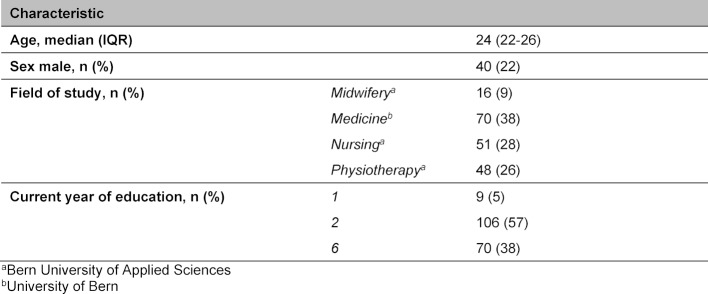
Demographics of participants for explanatory factor analysis (n=185), Bern, 2016

**Table 3:**
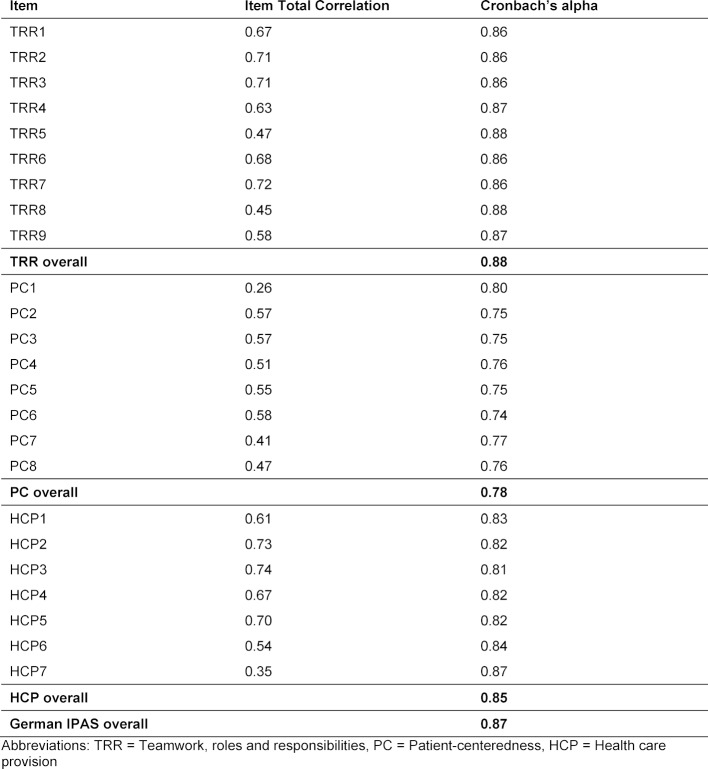
Item-Total Correlations and Cronbach’s alpha for the final German IPAS, Bern, 2016
