# Supplementary material for: The German interprofessional attitudes scale: translation, cultural adaptation, and validation
Source: GMS J Med Educ. 2020 Apr 15;37(3):Doc32. doi: 10.3205/zma001325 (PMC7291384; doi:10.3205/zma001325)
Supplement: Factor loading results of an exploratory factor analysis of the German Interprofessional Attitudes Scale, Bern, 2016 [file JME-37-32-s-001.pdf]

Attachment 1: Factor loading results of an exploratory factor analysis of the German Interprofessional Attitudes Scale, Bern, 2016

Item[a]	Scale and items	Factor loadings[b]		
	Eigenvalues	6.08	3.01	2.05
	Variance	4.38	3.82	2.95
		**Factor 1**	**Factor 2**	**Factor 3**
	Overall scale (3 subscales, 24 items)			
	**Teamarbeit, Funktionen und Verantwortlichkeiten**/Teamwork, roles and responsibilities			
**TRR1**	Interprofessionelles Lernen während des Studiums wird mir helfen, im Team besser zu arbeiten.<br>*Shared learning before graduation will help me become a better team worker.*	**0.74**	0.04	-0.02
**TRR2**	Gemeinsames Lernen wird mir helfen, positiv über andere Berufsgruppen zu denken.<br>*Shared learning will help me think positively about other professionals.*	**0.77**	0.07	0.11
**TRR3**	Lernen mit Studierenden anderer Gesundheitsberufe fördert meine Teamfähigkeit.<br>*Learning with other students will help me become a more effective member of a health care team.*	**0.75**	0.08	0.00
**TRR4**	Gemeinsames Lernen mit Studierenden anderer Gesundheitsberufe wird meine Fähigkeit verbessern, klinische Problemen zu verstehen.<br>*Shared learning with other health sciences students will increase my ability to understand clinical problems.*	**0.67**	0.09	0.12
**TRR5**	Patienten würden davon profitieren, wenn Studierende aus verschiedenen Gesundheitsberufen miteinander an der Lösung von Patientenproblemen arbeiten.<br>*Patients would ultimately benefit if health sciences students worked together to solve patient problems.*	**0.49**	0.26	0.07
**TRR6**	Gemeinsames Lernen mit Studierenden anderer Gesundheitsberufe wird mir helfen, besser mit Patienten und anderem Fachpersonal zu kommunizieren.<br>*Shared learning with other health sciences students will help me communicate better with patients and other professionals.*	**0.72**	0.18	0.06

Attachment 1 to: *Pedersen TH, Cignacco E, Meuli J, Habermann F, Berger-Estilita J, Greif R. The German interprofessional attitudes scale: Translation, cultural adaption, and validation. GMS J Med Educ. 2020;37(3):Doc32. DOI: 10.3205/001325*
Online available from: https://www.egms.de/en/journals/zma/2020-37/zma001325.shtml

Attachment 1: Factor loading results of an exploratory factor analysis of the German Interprofessional Attitudes Scale, Bern, 2016

**TRR7**	Ich würde es begrüssen, an Projekten in Kleingruppen mit Studierenden anderer Gesundheitsberufe zu arbeiten.<br>*I would welcome the opportunity to work on small group projects with other health sciences students.*	**0.77**	0.19	-0.09
**TRR8**[c]	Für Studierende von Gesundheitsberufen ist es nicht notwendig, miteinander zu lernen.<br>*It is not necessary for health sciences students to learn together.*	**-0.49**	-0.09	0.00
**TRR9**	Gemeinsames Lernen wird mir helfen, meine eigenen Grenzen zu erkennen.<br>*Shared learning will help me understand my own limitations.*	**0.61**	0.24	0.00
	**Patientenzentriertheit** / Patient-centeredness			
**PC1**	Der Aufbau von Vertrauen zwischen meinen Patienten und mir ist mir wichtig.<br>*Establishing trust with my patients is important to me.*	0.05	0.11	**0.31**
**PC2**	Es ist mir wichtig, meinen Patienten gegenüber Empathie zu zeigen.<br>*It is important for me to communicate compassion to my patients.*	0.06	0.06	**0.62**
**PC3**	Den Patienten als Individuum wahrzunehmen, ist für den Behandlungserfolg sehr wichtig.<br>*Thinking about the patient as a person is important in getting treatment right.*	-0.04	0.16	**0.67**
**PC4**	In meinem Beruf braucht man die Fähigkeit mit Patienten zu interagieren und auf Patienten einzugehen.<br>*In my profession, one needs skills in interacting and cooperating with patients.*	0.12	0.00	**0.62**
**PC5**	Es ist mir wichtig, die Sichtweise des Patienten zu verstehen.<br>*It is important for me to understand the patient's side of the problem.*	0.02	0.15	**0.59**
	**Interprofessionelle Vorurteile /** Interprofessional bias			
**IB1**	Fachpersonal / Studierende anderer Gesundheitsberufe haben mir gegenüber Vorurteile oder sind voreingenommen, aufgrund der Fachrichtung die ich studiere.<br>*Health professionals/students from other disciplines have prejudices or make assumptions about me because of the discipline I am studying.*	-0.04	0.16	-0.16

Attachment 1 to: *Pedersen TH, Cignacco E, Meuli J, Habermann F, Berger-Estilita J, Greif R. The German interprofessional attitudes scale: Translation, cultural adaption, and validation. GMS J Med Educ. 2020;37(3):Doc32. DOI: 10.3205/001325*
Online available from: https://www.egms.de/en/journals/zma/2020-37/zma001325.shtml

Attachment 1: Factor loading results of an exploratory factor analysis of the German Interprofessional Attitudes Scale, Bern, 2016

**IB2**	Ich habe Vorurteile oder bin voreingenommen gegenüber Fachpersonal / Studierenden anderer Gesundheitsberufe.<br>*I have prejudices or make assumptions about health professionals/students from other disciplines.*	-0.09	0.12	**-0.34**
**IB3**	Vorurteile gegen - oder Vorstellungen über Angehörige anderer Gesundheitsberufe behindern die Gesundheitsversorgung.<br>*Prejudices and assumptions about health professionals from other disciplines get in the way of delivery of health care.*	0.28	**0.33**	0.13
	**Kulturelle Vielseitigkeit und Ethik /** Diversity and Ethics			
**DE1**	Für medizinisches Fachpersonal ist es wichtig die Eigenheiten, Werte, Rollen, Verantwortlichkeiten und Expertise anderer Gesundheitsberufe zu respektieren.<br>*It is important for health professionals to respect the unique cultures, values, roles/responsibilities, and expertise of other health professions.*	0.12	**0.39**	0.25
**DE2**	Für medizinisches Fachpersonal ist es wichtig zu verstehen, wie man erfolgreich mit Menschen verschiedener kultureller Hintergründe kommuniziert.<br>*It is important for health professionals to understand what it takes to effectively communicate across cultures.*	0.08	0.29	**0.66**
**DE3**	Für medizinisches Fachpersonal ist es wichtig, bei der Patientenversorgung im Team, unter Einhaltung der Schweigepflicht, die Würde und Privatsphäre des Patienten zu wahren.<br>*It is important for health professionals to respect the dignity and privacy of patients while maintaining confidentiality in the delivery of team-based care.*	-0.01	0.14	**0.46**
**DE4**	Für medizinisches Fachpersonal ist es wichtig, dem Patienten unabhängig von seinem persönlichen Hintergrund eine optimale Behandlung zu gewähren (z.B. unabhängig von sexueller Orientierung, Geschlecht, Konfession, sozialer Stellung, ethnischer Herkunft, Immigrationsstatus oder Behinderung).<br>*It is important for health professionals to provide excellent treatment to patients regardless of their background (e.g., race, ethnicity, gender, sexual orientation, religion, class, national origin, immigration status, or ability).*	0.01	0.11	**0.54**
	**Stellung der Gesellschaft /** Community-centeredness			

Attachment 1: Factor loading results of an exploratory factor analysis of the German Interprofessional Attitudes Scale, Bern, 2016

**CC1**	Für medizinisches Fachpersonal ist es wichtig mit Entscheidungsträgern im Gesundheitswesen, aus Verwaltung und Politik zusammenzuarbeiten, um die Gewährleistung der Gesundheitsversorgung zu verbessern.<br>*It is important for health professionals to work with public health administrators and policy makers to improve delivery of health care.*	0.13	**0.67**	0.14
**CC2**	Für medizinisches Fachpersonal ist es wichtig an Projekten zur Verbesserung der öffentlichen Gesundheit mitzuarbeiten.<br>*It is important for health professionals to work on projects to promote community and public health.*	0.14	**0.78**	0.09
**CC3**	Für medizinisches Fachpersonal ist es wichtig an Gesetzgebungen, Verordnungen und politischen Strategien zur Verbesserung der Gesundheitsversorgung mitzuwirken.<br>*It is important for health professionals to work with the legislators to develop laws, regulations, and policies that improve health care.*	0.09	**0.84**	-0.02
**CC4**	Für medizinisches Fachpersonal ist es wichtig zusammen mit nicht klinisch-tätigen Berufsgruppen an einer Verbesserung der Effektivität der Gesundheitsversorgung zu arbeiten.<br>*It is important for health professionals to work with non-clinicians to deliver more effective health care.*	0.20	**0.71**	0.08
**CC5**	Für medizinisches Fachpersonal ist es wichtig sich neben der individuellen Patientenversorgung auch auf die Allgemeinheit zu konzentrieren, um eine effektive Gesundheitsversorgung anbieten zu können.<br>*It is important for health professionals to focus on populations and communities, in addition to individual patients, to deliver effective health care.*	0.22	**0.72**	0.09
**CC6**	Für medizinisches Fachpersonal ist es wichtig Fürsprecher für die Gesundheit von Patienten und der Allgemeinheit zu sein.<br>*It is important for health professionals to be advocates for the health of patients and communities.*	0.10	**0.57**	0.23

The items in both German and English. [a]TRR = Teamwork, roles and responsibilities, PC = Patient-centredness, IB = Interprofessional bias, DE = Diversity and Ethics, CC = Community-centeredness. [b]Factor loading > 0.30 for each item in bold. [c]TRR8 is reverse-coded for a positive correlation with items in the TRR subscale.

Attachment 1 to: *Pedersen TH, Cignacco E, Meuli J, Habermann F, Berger-Estilita J, Greif R. The German interprofessional attitudes scale: Translation, cultural adaption, and validation. GMS J Med Educ. 2020;37(3):Doc32. DOI: 10.3205/001325*
Online available from: https://www.egms.de/en/journals/zma/2020-37/zma001325.shtml
