# Supplementary material for: The German interprofessional attitudes scale: translation, cultural adaptation, and validation
Source: GMS J Med Educ. 2020 Apr 15;37(3):Doc32. doi: 10.3205/zma001325 (PMC7291384; doi:10.3205/zma001325)
Supplement: Final version German IPAS [file JME-37-32-s-002.pdf]

Attachment 2: Final version German IPAS

Interprofessional Attitudes Scale

INSELSPITAL
UNIVERSITÄTSSPITAL BERN
HOPITAL UNIVERSITAIRE DE BERNE
BERN UNIVERSITY HOSPITAL

Datum: ________________________

Alter: ________________________ Geschlecht: M □ W □

Studienrichtung/ Beruf im Gesundheitswesen:

____

Aktuelles Studienjahr / Berufsjahre : ______________

Vergangene Teilnahmen an interprofessionellen Veranstaltungen: Ja □ Nein □

Wenn Ja, welche: ____

Studien ID:

1 = Trifft nicht zu
2 = Trifft eher nicht zu
3 = Teils-teils
4 = Trifft eher zu
5 = Trifft zu

Bitte zutreffende Aussage ankreuzen:

1. Teamarbeit, Funktionen und Verantwortlichkeiten

**1**	Interprofessionelles Lernen während des Studiums wird mir helfen, im Team besser zu arbeiten.	1 □ 2 □ 3 □ 4 □ 5 □
**2**	Gemeinsames Lernen wird mir helfen positiv über andere Berufsgruppen zu denken.	1 □ 2 □ 3 □ 4 □ 5 □
**3**	Lernen mit Studierenden anderer Gesundheitsberufe fördert meine Teamfähigkeit.	1 □ 2 □ 3 □ 4 □ 5 □
**4**	Gemeinsames Lernen mit Studierenden anderer Gesundheitsberufe wird meine Fähigkeit verbessern, klinische Problemen zu verstehen.	1 □ 2 □ 3 □ 4 □ 5 □
**5**	Patienten würden davon profitieren, wenn Studierende aus verschiedenen Gesundheitsberufen miteinander an der Lösung von Patientenproblemen arbeiten.	1 □ 2 □ 3 □ 4 □ 5 □
**6**	Gemeinsames Lernen mit Studierenden anderer Gesundheitsberufe wird mir helfen, besser mit Patienten und anderem Fachpersonal zu kommunizieren.	1 □ 2 □ 3 □ 4 □ 5 □
**7**	Ich würde es begrüssen, an Projekten in Kleingruppen mit Studierenden anderer Gesundheitsberufe zu arbeiten.	1 □ 2 □ 3 □ 4 □ 5 □
**8**	Für Studierende von Gesundheitsberufen ist es nicht notwendig miteinander zu lernen.	1 □ 2 □ 3 □ 4 □ 5 □
**9**	Gemeinsames Lernen wird mir helfen meine eigenen Grenzen zu erkennen.	1 □ 2 □ 3 □ 4 □ 5 □

Bitte wenden!

Attachment 2 to: *Pedersen TH, Cignacco E, Meuli J, Habermann F, Berger-Estilita J, Greif R. The German interprofessional attitudes scale: Translation, cultural adaption, and validation. GMS J Med Educ. 2020;37(3):Doc32. DOI: 10.3205/001325*
Online available from: https://www.egms.de/en/journals/zma/2020-37/zma001325.shtml

2. Patientenzentriertheit

1	Der Aufbau von Vertrauen zwischen meinen Patienten und mir ist mir wichtig.	1 □ 2 □ 3 □ 4 □ 5 □
2	Es ist mir wichtig, meinen Patienten gegenüber Empathie zu zeigen.	1 □ 2 □ 3 □ 4 □ 5 □
3	Den Patienten als Individuum wahrzunehmen, ist für den Behandlungserfolg sehr wichtig.	1 □ 2 □ 3 □ 4 □ 5 □
4	In meinem Beruf braucht man die Fähigkeit mit Patienten zu interagieren und auf Patienten einzugehen.	1 □ 2 □ 3 □ 4 □ 5 □
5	Es ist mir wichtig, die Sichtweise des Patienten zu verstehen.	1 □ 2 □ 3 □ 4 □ 5 □
6	Für medizinisches Fachpersonal ist es wichtig zu verstehen, wie man erfolgreich mit Menschen verschiedener kultureller Hintergründe kommuniziert.	1 □ 2 □ 3 □ 4 □ 5 □
7	Für medizinisches Fachpersonal ist es wichtig bei der Patientenversorgung im Team, unter Einhaltung der Schweigepflicht, die Würde und Privatsphäre des Patienten zu wahren.	1 □ 2 □ 3 □ 4 □ 5 □
8	Für medizinisches Fachpersonal ist es wichtig dem Patienten unabhängig von seinem persönlichen Hintergrund eine optimale Behandlung zu gewähren (z.B. unabhängig von sexueller Orientierung, Geschlecht, Konfession, sozialer Stellung, ethnischer Herkunft, Immigrationsstatus oder Behinderung).	1 □ 2 □ 3 □ 4 □ 5 □

3. Gesundheitsversorgung

Für medizinisches Fachpersonal ist es wichtig…

1	… mit Entscheidungsträgern im Gesundheitswesen aus Verwaltung und Politik zusammenzuarbeiten, um die Gewährleistung der Gesundheitsversorgung zu verbessern.	1 □ 2 □ 3 □ 4 □ 5 □
2	… an Projekten zur Verbesserung der öffentlichen Gesundheit mitzuarbeiten.	1 □ 2 □ 3 □ 4 □ 5 □
3	… an Gesetzgebungen, Verordnungen und politischen Strategien zur Verbesserung der Gesundheitsversorgung mitzuwirken.	1 □ 2 □ 3 □ 4 □ 5 □
4	… zusammen mit nicht klinisch-tätigen Berufsgruppen an einer Verbesserung der Effektivität der Gesundheitsversorgung zu arbeiten.	1 □ 2 □ 3 □ 4 □ 5 □
5	… sich neben der individuellen Patientenversorgung auch auf die Allgemeinheit zu konzentrieren, um eine effektive Gesundheitsversorgung anbieten zu können.	1 □ 2 □ 3 □ 4 □ 5 □
6	… Fürsprecher für die Gesundheit von Patienten und der Allgemeinheit zu sein.	1 □ 2 □ 3 □ 4 □ 5 □
7	… die Eigenheiten, Werte, Rollen, Verantwortlichkeiten und Expertise anderer Gesundheitsberufe zu respektieren.	1 □ 2 □ 3 □ 4 □ 5 □

Attachment 2 to: *Pedersen TH, Cignacco E, Meuli J, Habermann F, Berger-Estilita J, Greif R. The German interprofessional attitudes scale: Translation, cultural adaption, and validation. GMS J Med Educ. 2020;37(3):Doc32. DOI: 10.3205/001325*
Online available from: https://www.egms.de/en/journals/zma/2020-37/zma001325.shtml
